# New Perspectives in Cardiac and Vascular Diseases

**DOI:** 10.3390/biomedicines13061377

**Published:** 2025-06-04

**Authors:** Pietro Scicchitano, Francesco Massari

**Affiliations:** Cardiology Section, Hospital “F. Perinei” ASL BA, 70022 Altamura, Bari, Italy; francesco.massari@asl.bari.it

Understanding the physiopathology of cardiac and vascular diseases presents a challenge to clinicians due to their intricate biochemical and molecular mechanisms [1,2]. Clarity is essential for the characterization and development of pharmaceutical and non-pharmaceutical compounds that have a positive impact on the clinical course of various diseases [3,4].

Cardiovascular diseases (CVDs) remain a predominant global health burden, responsible for nearly one-third of all deaths worldwide [5]. The rising prevalence of hypertension [6,7], metabolic disorders [8], arrhythmogenic conditions [9,10], and heart failure underscores the urgent need for multidisciplinary approaches to research and clinical management [5,11].

This second edition of the Special Issue, titled “Cardiac and Vascular Diseases: Pathogenesis, Pharmacological Treatments, Advances in Therapies”, features a collection of high-quality, insightful original articles, reviews, and case studies that span the continuum of cardiovascular disease—from molecular mechanisms to clinical applications.

One of the most striking contributions in this Issue is the study by He et al. [12], in which they demonstrate the cardioprotective potential of baicalin, a natural flavonoid extracted from *Scutellaria baicalensis* [13]. Using both in vivo and in vitro models of heart failure, the authors show that baicalin ameliorates cardiac hypertrophy and fibrosis by inhibiting the p85α subunit of the PI3K signaling pathway. This work not only uncovers a new pharmacological target in the treatment of heart failure but also reinforces the emerging role of phytochemicals in cardiovascular therapeutics.

In the field of pharmacovigilance, Mitkova et al. [14] provide valuable real-world data from the Bulgarian population regarding adverse drug reactions (ADRs) to commonly prescribed cardiovascular drugs, including ACE inhibitors, sartans, and statins. Their analysis reveals a substantial frequency of serious ADRs—particularly involving valsartan and rosuvastatin—highlighting the ongoing need for active surveillance and personalized medicine approaches, especially in polypharmacy and elderly populations.

The Issue also examines the critical role of diagnostic innovation in cardiovascular prevention. Kosowski and Aleksandrowicz [15] review the clinical significance of a hypertensive response to exercise (HRE), an under-recognized marker of early cardiovascular dysfunction. Their synthesis of current evidence suggests that HRE correlates with future hypertension, left ventricular hypertrophy, and increased cardiovascular risk—even in asymptomatic individuals. The authors advocate for broader clinical use of exercise stress testing not just for ischemia detection but also as a prognostic tool in preventive cardiology.

Within the realm of thrombosis, Fioretti et al. [16] present a comprehensive review of peripherally inserted central catheter (PICC)-associated vein thrombosis in oncology patients. With venous thromboembolism recognized as the second leading cause of death in cancer [17], this review offers practical recommendations for thromboprophylaxis and device management, emphasizing the complexity of balancing oncologic and cardiovascular care.

The intersection of infectious disease and cardiovascular risk is explored by Rajewski et al. [18], who report that the Hepatitis C virus (HCV) infection is not an independent cardiovascular risk factor in young adults. This nuanced finding is especially relevant in the era of successful antiviral therapy and may inform screening strategies in younger, low-risk populations.

Furthering our understanding of inherited cardiomyopathies, Młynarska et al. [19] focus on hypertrophic cardiomyopathy (HCM) and the emerging role of mavacamten, a selective cardiac myosin inhibitor [20]. Their review synthesizes the evidence for mavacamten as a groundbreaking therapy capable of modulating the underlying hypercontractility in HCM, potentially redefining treatment algorithms for this complex condition.

Lastly, Wierciak-Rokowska et al. [21] report a rare case of upper vascular thoracic outlet syndrome (TOS), highlighting the diagnostic challenges posed by this uncommon vascular entity. Their case study reinforces the value of individualized diagnostic pathways and collaborative management strategies in vascular medicine.

Together, these contributions reflect the evolving complexity of cardiovascular disease research and clinical care. The impact of new drugs and therapeutics is crucial to human health, given the significant influence of cardiovascular diseases on prognosis [22], quality of life [23], and social costs [24].

From novel drug targets and real-world pharmacovigilance to early disease detection and precision therapeutics, this Special Issue affirms the necessity of a multidisciplinary and translational approach. The integration of molecular insights with clinical realities will be key to overcoming the persistent challenges posed by cardiac and vascular diseases.

We hope that the research presented in this Issue inspires continued innovation, collaboration, and personalized care in the fight against cardiovascular disease.

## References

[1] Li Y., Du J., Deng S., Liu B., Jing X., Yan Y., Liu Y., Wang J., Zhou X., She Q. (2024). The molecular mechanisms of cardiac development and related diseases. Signal Transduct. Target. Ther..

[2] Scott J. (2004). Pathophysiology and biochemistry of cardiovascular disease. Curr. Opin. Genet. Dev..

[3] Gromo G., Mann J., Fitzgerald J.D. (2014). Cardiovascular drug discovery: A perspective from a research-based pharmaceutical company. Cold Spring Harb. Perspect. Med..

[4] Hong C.C. (2022). The grand challenge of discovering new cardiovascular drugs. Front. Drug Discov..

[5] Cheng Q., Zhou S., Zhong H., Wang Z., Liu C., Sun J., Deng J. (2025). Global, regional, and national burden and risk factors of ischemic heart disease, 1990–2021: An analysis of the global burden of disease study. Front. Public Health.

[6] Mills K.T., Stefanescu A., He J. (2020). The global epidemiology of hypertension. Nat. Rev. Nephrol..

[7] NCD Risk Factor Collaboration (NCD-RisC) (2021). Worldwide trends in hypertension prevalence and progress in treatment and control from 1990 to 2019: A pooled analysis of 1201 population-representative studies with 104 million participants. Lancet.

[8] Gurka M.J., Filipp S.L., DeBoer M.D. (2018). Geographical variation in the prevalence of obesity, metabolic syndrome, and diabetes among US adults. Nutr. Diabetes.

[9] Sharma A., Bosman L.P., Tichnell C., Nanavati J., Murray B., Nonyane B.A.S., Tandri H., Calkins H., James C.A. (2022). Arrhythmogenic Right Ventricular Cardiomyopathy Prevalence and Arrhythmic Outcomes in At-Risk Family Members: A Systematic Review and Meta-Analysis. Circ. Genom. Precis. Med..

[10] Agbaedeng T.A., Roberts K.A., Colley L., Noubiap J.J., Oxborough D. (2022). Incidence and predictors of sudden cardiac death in arrhythmogenic right ventricular cardiomyopathy: A pooled analysis. Europace.

[11] GBD 2019 Acute and Chronic Care Collaborators (2025). Characterising acute and chronic care needs: Insights from the Global Burden of Disease Study 2019. Nat. Commun..

[12] He L., Zhu M., Yin R., Dai L., Chen J., Zhou J. (2025). Baicalin Mitigates Cardiac Hypertrophy and Fibrosis by Inhibiting the p85a Subunit of PI3K. Biomedicines.

[13] Jiang M., Li Z., Qin X., Chen L., Zhu G. (2025). Regulatory Role of Flavonoid Baicalin from *Scutellaria baicalensis* on AMPK: A Review. Am. J. Chin. Med..

[14] Mitkova Z., Dimova A., Petrova G., Dimitrova M. (2024). Adverse Drug Reactions of Cardiovascular Classes of Medicines-Data for Bulgarian Population. Biomedicines.

[15] Kosowski W., Aleksandrowicz K. (2024). Hypertensive Response to Exercise as an Early Marker of Disease Development. Biomedicines.

[16] Fioretti A.M., Scicchitano P., La Forgia D., De Luca R., Campello E., Tocchetti C.G., Di Nisio M., Oliva S. (2025). Prevention of Peripherally Inserted Central Catheter (PICC)-Associated Vein Thrombosis in Cancer: A Narrative Review. Biomedicines.

[17] Donnellan E., Khorana A.A. (2017). Cancer and Venous Thromboembolic Disease: A Review. Oncologist.

[18] Rajewski P., Pawłowska M., Kozielewicz D., Dybowska D., Olczak A., Cieściński J. (2024). Hepatitis C Infection Is Not a Cardiovascular Risk Factor in Young Adults. Biomedicines.

[19] Młynarska E., Radzioch E., Dąbek B., Leszto K., Witkowska A., Czarnik W., Jędraszak W., Rysz J., Franczyk B. (2024). Hypertrophic Cardiomyopathy with Special Focus on Mavacamten and Its Future in Cardiology. Biomedicines.

[20] Maheta D., Agrawal S.P., Aronow W.S. (2025). Advances in the management of obstructive hypertrophic cardiomyopathy: The role of mavacamten. Expert. Opin. Pharmacother..

[21] Wierciak-Rokowska A., Sliwka A., Maga M., Gajda M., Bogucka K., Kaczmarczyk P., Maga P. (2024). Upper Vascular Thoracic Outlet Syndrome: A Case Study. Biomedicines.

[22] Ma H., Wang X., Xue Q., Li X., Liang Z., Heianza Y., Franco O.H., Qi L. (2023). Cardiovascular Health and Life Expectancy Among Adults in the United States. Circulation.

[23] Chatzinikolaou A., Tzikas S., Lavdaniti M. (2021). Assessment of Quality of Life in Patients with Cardiovascular Disease Using the SF-36, MacNew, and EQ-5D-5L Questionnaires. Cureus.

[24] Lui J.N.M., Williams C., Keng M.J., Hopewell J.C., Sammons E., Chen F., Gray A., Bowman L., Landray S.M.J., Mihaylova B. (2023). Impact of New Cardiovascular Events on Quality of Life and Hospital Costs in People with Cardiovascular Disease in the United Kingdom and United States. J. Am. Heart Assoc..

